# MRI structural alterations in schizophrenia patients with prominent first rank symptoms

**DOI:** 10.1192/j.eurpsy.2025.1667

**Published:** 2025-08-26

**Authors:** A. S. Tomyshev, M. M. Magomedagaev, A. Y. Komarova, A. N. Dudina, G. P. Kostyuk, R. V. Dmitry, I. S. Lebedeva

**Affiliations:** 1 Laboratory of neuroimaging and multimodal analysis, Mental Health Research Center; 2 Department of Psychiatry and Psychosomatics, I.M. Sechenov First Moscow State Medical University; 3 Mental-health Clinic No.1 named after N.A. Alexeev; 4 Department of borderline mental pathology and psychosomatic disorders, Mental Health Research Center, Moscow, Russian Federation

## Abstract

**Introduction:**

There is an evidence that the presence of first rank symptoms (FRS) in psychotic patients is associated with structural brain alterations and that the FRS severity is correlated with abnormal brain functioning. However, whether the severity of first rank delusions (FRD) or first rank hallucinations (FRH) correlates with structural alterations remains unclear.

**Objectives:**

We aimed at exploring correlations of FRS severity with structural brain alterations in schizophrenia patients with prominent first rank symptoms.

**Methods:**

Twenty one right-handed patients (21.2-47.6 years, mean age 35.2±8.8, 2 females) with schizophrenia, presenting with prominent FRS and 21 one-to-one matched healthy controls (21.4-47.7 years, mean age 34.6±9.0, 2 females) underwent structural MRI at 3T scanner. MRI images were processed via FreeSurfer 6.0 to quantify cortical thickness and volumes for subcortical and brainstem (midbrain, pons, superior cerebellar peduncle and medulla) structures. The presence of FRS were diagnosed by professional psychiatrist (M.M.) using clinical interview and clinical-psychopathological method, severity of FRD and FRH were assessed with PANSS (P1 and P3 items accordingly). PANSS total for all patients: 88.1±20.6; PASNSS positive: 23.7±5.2 (P1: 4.9±1.3; P3: 4.6±1.3); PASNSS negative: 21.9±8.7.

**Results:**

Compared to healthy controls, patients with FRS showed widespread cortical gray matter thickness reductions (Image 1, A), and decreased volumes of hippocampus, amygdala, thalamus, caudate and nucleus accumbens bilaterally (Image 1, B). Patients with FRS also showed decreased volumes of whole brainstem and all of its substructures (midbrain, pons, superior cerebellar peduncle and medulla: Cohen’s *d* from −0.74 to −1.2).

No correlations between structural alterations and severity of FRD or FRH (PANSS P1, P3, P1+P3) were found.

Image 1. A: Clusters of decreased cortical thickness according to atlas of Desikan et al. (2006) in patients with FRS compared to healthy controls. B: Decreased volumes of subcortical structures in patients with FRS compared to healthy controls (nucleus accumbens are not shown).

**Image 1:**

**Figure:**
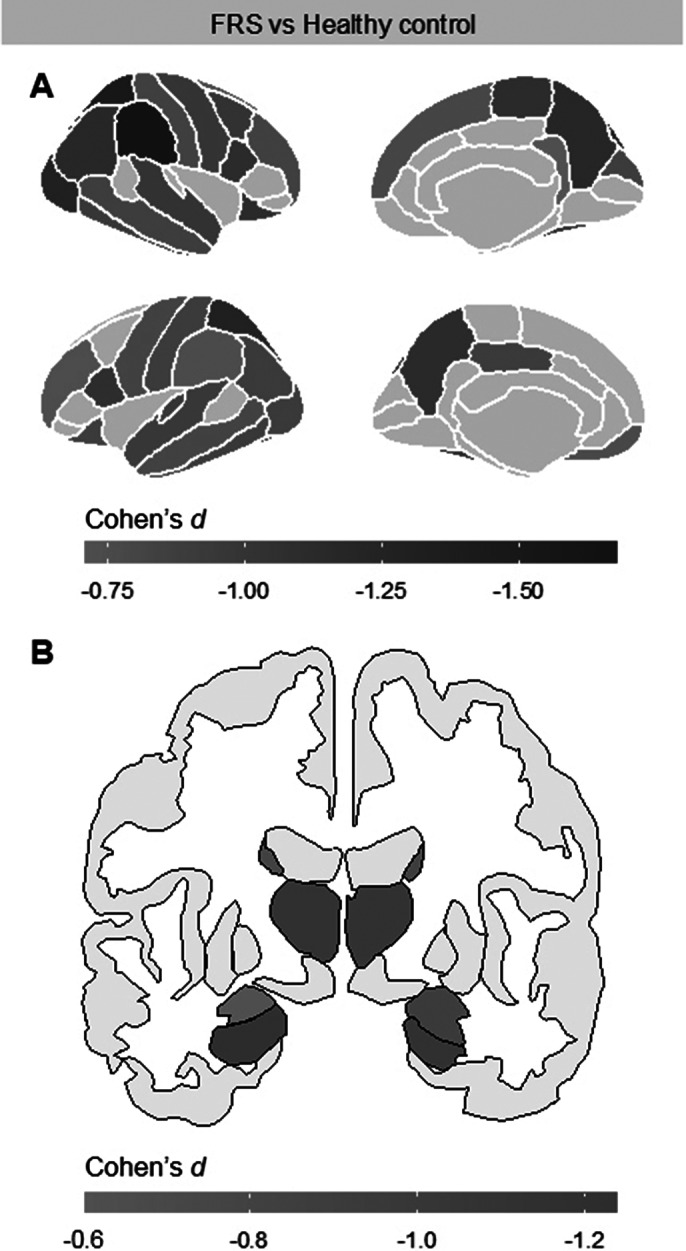

**Conclusions:**

The results suggest that schizophrenia patients with prominent FRS have a pronounced widespread cortical and subcortical structural alterations consistent with findings in general schizophrenia research. However, the lack of correlations with clinical scores do not allow to conclude definitely whether the revealed structural abnormalities underlies FRD or FRH, which should be elucidated via further research.

**Disclosure of Interest:**

None Declared

